# The Skin—A Common Pathway for Integrating Diagnosis and Management of NTDs

**DOI:** 10.3390/tropicalmed3030101

**Published:** 2018-09-10

**Authors:** David John Chandler, Lucinda Claire Fuller

**Affiliations:** Dermatology Department, Brighton General Hospital, East Sussex BN2 3EW, UK; claire.fuller@nhs.net

**Keywords:** integration, neglected tropical diseases, disease mapping, mass drug administration, morbidity management

## Abstract

Many of the neglected tropical diseases (NTDs) have major skin manifestations. These skin-related NTDs or ‘skin NTDs’ cause significant morbidity and economic hardship in some of the poorest communities worldwide. We draw attention to the collective burden of skin disease and suggest that the skin be used as a platform for the integration of control activities for NTDs. The opportunities for integration are numerous, ranging from diagnosis and disease mapping to mass drug administration and morbidity management. The dermatology community has an important role to play, and will be expected to support research and control activities globally.

## 1. Introduction

Skin and subcutaneous diseases account for substantial morbidity worldwide, with a high burden of disease in resource-poor tropical regions [1,2]. Neglected tropical diseases (NTDs) are a group of communicable diseases that are present throughout the tropical and subtropical regions of the world, where they affect the poorest and most marginalised communities. The World Health Organization (WHO) portfolio of NTDs comprises a list of 20 diseases that affect more than 2 billion people worldwide [3].

Eighteen of the twenty NTDs on the WHO list have recognised skin manifestations. In some cases, involvement of the skin is the primary manifestation of the disease; examples include Buruli ulcer, leprosy, scabies, and mycetoma. In other cases, skin involvement may be an associated clinical feature, such as the urticarial rash that can occur in schistosomiasis, soil-transmitted helminthiases, and foodborne trematodiases. The only NTDs that do not have skin manifestations are rabies and trachoma. In addition, several other diseases affecting the skin, whilst not prioritised on the WHO list, are recognised as NTDs; examples include cutaneous larva migrans, impetigo and cellulitis, podoconiosis, tropical ulcer, and tungiasis.

These skin-related NTDs or ‘skin NTDs’ frequently result in physical impairment and disfigurement, which can lead to life-long disability, inability to work, social stigmatisation, and discrimination. Examples of disability include permanent nerve damage and limb deformity in leprosy, advanced lymphoedema and hydrocele in lymphatic filariasis, severe itching and hanging groin in onchocerciasis, and amputation in cases of severe mycetoma. However, the true global burden of many skin NTDs is poorly understood. Data from the Global Burden of Disease Study (GBD) 2013 identified lymphatic filariasis and scabies as the 4th and 5th leading causes of morbidity out of all NTDs, resulting in 2.02 million and 1.71 million years lived with disability (YLD), respectively; however, these estimates are likely to be conservative [1,3,4].

Fungal diseases have been neglected for many years, with very little funding made available for research and control activities [5]. Consequently, high-quality data on the epidemiology and associated morbidity of superficial and deep mycoses do not exist, which makes it difficult to advocate for increased funding. The addition of this disease group to the WHO list of NTDs in 2017 should assist in raising its profile in the global health agenda; the same applies for scabies, which joined the WHO list at the same time.

The impoverishing effects of NTDs are amplified in poor and vulnerable communities; physical labour is often the main source of income, and social protection mechanisms (insurance against ill-health and disability) are not accessible [6]. In rural India, households with a family member affected by leprosy can incur catastrophic health expenditure, mostly as a result of loss of income due to chronic ill-health [7,8]. Significant health expenditure and productivity loss has also been demonstrated for other skin NTDs including podoconiosis and leishmaniasis [9,10,11].

Historically, disease control activities have operated within vertical programs that were set up to deal with specific priority diseases; however, these were resource intensive and inefficient. Integration offers a cost-effective and efficient approach for tackling groups of related conditions. Skin NTDs are frequently co-endemic in low-resource settings, and many of the available treatments and management strategies are beneficial for more than one condition; the skin has therefore been proposed as a common pathway to enhance the control of these neglected diseases. 

From the growing literature on this subject, two key articles are worth highlighting. Engelman et al. discuss in detail many of the opportunities and challenges of integration [12]. In a paper that followed shortly after, an expert panel develop some of these ideas and offer practical advice on how to approach integration of surveillance and management activities for the skin NTDs [13].

The purpose of this review is to provide an update on the subject of skin NTDs and the opportunities for integration. We highlight examples where integration has been successful and draw attention to research questions that remain unanswered.

## 2. Methodology

We considered all types of publication and study design in our literature search; articles not written in English were excluded. We searched the following databases to July 2018: MEDLINE (from 1946), Embase (from 1947), and Allied & Complementary Medicine (from 1985). Core search terms included but were not limited to ‘neglected tropical diseases’, ‘skin NTDs’, ‘integration’, ‘diagnosis’, ‘disease mapping’, ‘morbidity management’, and ‘mass drug administration’. We communicated with researchers active in this field to identify unpublished data and used the bibliographies of articles to expand our search.

## 3. Integrated Approach to Skin NTDs

An integrated approach to tackling skin NTDs offers many benefits, and the dermatology community has an important role to play in guiding this process. Integration in this context refers to combining activities for two or more diseases at the same time and in the same communities. Integration could be beneficial for several activities, from diagnosis and mapping to treatment and education, and these are discussed in more detail below.

## 4. Diagnosis

Skin disease is very common in resource-poor regions. Individuals frequently have more than one skin NTD, in addition to other, much more common skin conditions. Reliable diagnostic tests do not exist for many NTDs; therefore, diagnosis is based on clinical examination.

Clinical signs of skin NTDs are diverse; however, the diagnostic process can be simplified with the use of syndrome-based assessment tools. WHO have produced a training guide titled ‘Recognising neglected tropical diseases through changes on the skin’, which is intended to support front-line health workers without specialist knowledge of dermatology to detect skin changes suggestive of an underlying NTD [14]. Four major skin changes are identified: lumps, ulcers, swollen limbs, and patches. For each skin change there is a diagnostic flow-chart which guides the user towards a likely diagnosis and a decision on treatment or further assessment (referral).

The success of training health workers to identify skin NTDs has been demonstrated at the community level previously. Limited training of primary health care workers in Mali, focusing on leprosy and common skin diseases, resulted in significant improvements in diagnostic accuracy, appropriateness of referrals, and ability to correctly examine neurological (sensory) function, 12–18 months after the training was completed [15]. Traditional healers and those practising alternative systems of medicine are widely available in many African countries and throughout India, particularly in the rural communities, where they are easy to access and often a preferred source of treatment for health problems including leprosy [16]. Several studies have shown that it is both feasible and cost-effective to recruit traditional healers and train them to detect the signs of leprosy and refer suspected cases to specialist leprosy services [17,18,19]. Enhancing new case detection and avoiding delays to treatment are important goals of leprosy control. However, a recent article by Darlong et al. conducted in West Bengal, India, highlights delays to diagnosis in children with multibacillary leprosy, resulting in a significant number of cases of grade 2 disability at presentation [20]. All of the affected children in the study had leprosy-specific symptoms for more than 6 months before diagnosis, many of which involved visible skin changes such as ulceration or a skin patch. Strategies are needed to increase detection of these cases, including educational interventions that encourage earlier presentation to health services.

Integration of control activities for skin NTDs might offer further benefit by providing an operational framework through which other common (non-NTD) skin conditions can be detected and treated; however, the challenge this will provide should not be underestimated, and health workers in this setting should receive training in general dermatology. For example, it is important to appreciate that a hypopigmented skin patch has several causes, many of which are much more likely than leprosy, such as pityriasis versicolor, pityriasis alba, and achromic naevus. The importance of this particular point has been highlighted recently by several authors [21,22]. In a survey of skin NTDs, involving more than 13,000 schoolchildren aged 5 to 15 in Côte d’Ivoire, West Africa, the overall prevalence of skin disease was 25.6% [21]. Superficial mycoses were by far the most common diagnoses, and the majority of children were diagnosed with 2 or more skin conditions. Of 986 children examined by a dermatologist, the skin NTDs detected included one case of leprosy and 36 cases of scabies. Barogui and colleagues report similar findings from their experience of integrated control activities against Buruli ulcer, leprosy, and yaws in Benin, West Africa [22]. Trained health workers and community health volunteers identified 1106 patients with skin disease. Skin NTDs included only 15 cases of Buruli ulcer and 3 cases of leprosy. There were 185 suspected cases of yaws; however, these all tested negative using the point-of-care Dual Path Platform (DPP) syphilis assay.

An integrated approach to diagnosis offers the opportunity to detect multiple conditions in a single encounter. Vertical disease programs have a narrow focus and do not necessarily allow for detection and treatment of other important co-existent skin conditions. The majority of skin disease is accounted for by a few common conditions; these include bacterial skin infections (impetigo, boils), superficial mycoses, non-infective conditions such as eczema, and traumatic lesions. Algorithms for detecting and managing these skin conditions have been validated and applied in various settings and these could be applied in future integration efforts [23,24]. It would be appropriate and realistic for community-based health workers with limited dermatological training to focus on identifying and managing these common skin conditions for which relatively simple and effective treatments are available.

## 5. Disease Mapping and Surveillance

High-quality epidemiological data are lacking for many skin NTDs, and it is important for the success of integrated control efforts that this is addressed early. Disease mapping and identification of geographic areas of co-endemicity are important to determine disease burden and direct further research and control strategies. This step must take place before integrated management and control programmes can be planned and resources allocated. Consensus criteria for diagnosis of skin NTDs should be used, such as those available for podoconiosis [25] and, more recently, scabies [26].

Disease mapping refers to the collection of georeferenced data used to visualise the distribution and prevalence of a disease in space and time [27]. Maps are developed using a geographical information system (GIS) which allows storage, analysis, and presentation of spatial data. The African Programme for Onchocerciasis Control (APOC) was one of the earliest GIS-based mapping initiatives and has been used throughout Africa to identify priority areas for community treatment with ivermectin; APOC has also been used to map the distribution of *Loa loa* and identify areas of high prevalence where there is an increased risk of developing serious adverse events following treatment with ivermectin [28].

Integration of mapping and surveillance activities could be better suited to certain diseases, particularly those that share demographic and geographic preferences. Diseases causing lymphoedema provide a good example of where integration is suitable and where integrated mapping has been successful. Lymphoedema in the tropics has two main causes—lymphatic filariasis (LF), which is caused by infection with a parasitic nematode, predominantly *Wuchereria bancrofti*, and podoconiosis, which is caused by prolonged contact with irritant red clay soils derived from volcanic deposits.

Sime and colleagues describe their experience of the first integrated mapping of LF and podoconiosis, which captured almost 130,000 people from 1315 communities across 659 districts of Ethiopia during a 3-month period [29]. LF cases were identified by taking a blood sample and testing for circulating *Wuchereria bancrofti* antigen using an immunochromatographic card test (ICT). Podoconiosis was diagnosed clinically by excluding other causes of lymphoedema such as rheumatic heart disease, leprosy, and onchocerciasis. Smartphones were used for data collection; data were entered in real time and geographic coordinates were captured using an inbuilt geographic positioning system (GPS) function. The authors reported that using an integrated process was cost-effective and enabled rapid mapping of a larger geographical area than would otherwise have been achieved by using an individual disease survey. Recently, an integrated mapping exercise was conducted in Ethiopia, in 20 districts co-endemic for LF and podoconiosis, to identify all cases of lymphoedema [30]. More than 26,000 cases of lymphoedema and/or hydrocele were identified, and the majority of cases (>95%) had leg lymphoedema only, which could be due to LF or podoconiosis. These results will inform the planning and implementation of morbidity management and disability prevention (MMDP) services which are equally beneficial in both LF and podoconiosis.

Integrated mapping is more cost-effective than disease-specific mapping and allows for more precise mapping of co-endemic diseases and related conditions. In the Solomon Islands, Mason et al. conducted a cross-sectional study to assess the prevalence of scabies and impetigo [31], recruiting more than 1900 patients to the study; this was achieved by integrating data collection with an existing epidemiological study investigating clinical and serological markers of yaws [32].

## 6. Treatment

The last two decades have seen significant achievements in the control of a number of important tropical infections, including significant reductions in the prevalence of diseases such as lymphatic filariasis, guinea worm, and leprosy [33,34]. The successes of energetic vertical programmes are acknowledged; however, resource constraints have led to an increasing emphasis on integrating control activities.

Many of the opportunities for integrated treatment of skin NTDs exist for conditions that are suitable for control using mass drug administration (MDA), and, in particular, those that exhibit significant geographical overlap. Of note, seven of the most prevalent and debilitating NTDs have been the target of control efforts using integrated MDA; these include lymphatic filariasis, onchocerciasis, schistosomiasis, trachoma, and the three major soil-transmitted helminth infections (ascariasis, trichuriasis, and hookworm infection) [35]. Partnership between public and private organisations, multinational drug companies, and national ministries of health enabled the distribution of ‘rapid-impact’ packages of drugs throughout Africa, providing treatment for all seven of the NTDs at low cost. Ivermectin is highly effective at killing microfilariae in lymphatic filariasis and onchocerciasis, and was one of the drugs included in the rapid-impact package. Mostly due to drug company donations, it was possible to achieve a unit cost of US$0.40 per person annually, and in some cases even lower [35]. Data from the Global Burden of Disease (GBD) Study 2016 have shown considerable reductions in disease prevalence and burden (measured in DALYs) for six of the seven NTDs targeted by ‘rapid-impact’ integrated MDA over the last decade [36].

Ivermectin is widely recognised as a broad-spectrum antiparasitic drug, capable of killing parasites both inside and outside the body. It is particularly useful for the control of several skin NTDs. It is the treatment of choice for strongyloidiasis, and it is highly effective against scabies, pediculosis capitis, and cutaneous larva migrans [37,38]. Collateral benefits of ivermectin MDA have been observed in various settings, for example, in the lymphatic filariasis and onchocerciasis control programmes, where it was responsible for reducing the prevalence of tungiasis and scabies [38,39,40]. Ivermectin is also an effective treatment for gnathostomiasis and soil-transmitted helminthiasis. Improving access to low-cost generic ivermectin and broadening the licensed indications for use are some of the challenges to be addressed in order to realise the full public health potential of this drug. Studies of ivermectin MDA for scabies control have shown that the drug has a very good safety profile [41]. Although robust safety data in pregnant women and children under 5 years of age are lacking, the few published studies have failed to identify any difference in birth defects or developmental status in children born to women inadvertently treated with ivermectin during pregnancy [42,43,44]. Monitoring of existing ivermectin MDA programmes for safety outcomes will add to our understanding.

Moxidectin is a second-generation macrocyclic lactone related to ivermectin. It has demonstrated superior efficacy over ivermectin for the treatment of onchocerciasis [45], and recently received FDA approval (June 2018) for patients 12 years of age and older. In addition, moxidectin looks promising as a treatment for scabies. It has enhanced bioavailability (half-life of over 20 days, compared with 14 h for ivermectin) and better skin retention, meaning that a single-dose regimen may be possible [46]. Moxidectin is superior to ivermectin against the scabies mite in vitro [47]; however, clinical studies are required in order to establish suitable doses and evaluate efficacy in cases of scabies.

Integrated treatment programs can be particularly advantageous for diseases that can be treated using a single medication, as this removes the possibility of drug interactions and adverse safety events that may arise from coadministration of multiple drugs. For example, both yaws and trachoma can be targeted by mass administration of azithromycin, although the recommended doses for each are different—20 mg/kg for trachoma, and 30 mg/kg for yaws. However, a study conducted in the Solomon Islands showed that the lower dose (20 mg/kg) was effective against yaws; no active cases of yaws were identified following a single round of treatment using the lower dose in a previously endemic population [32]. This supports the argument for merging and simplifying treatment algorithms where possible and integrating mass treatment of these conditions.

On the other hand, mass treatment using more than one drug has the benefit of targeting a greater number of diseases and therefore delivering a greater impact to the health of the affected population. The effectiveness of ivermectin against highly symptomatic skin diseases such as scabies is an important factor in promoting adherence with further rounds of treatment in MDA programmes [48]; administering ivermectin together with other drugs against co-endemic skin NTDs would likely have a positive effect on overall compliance. Ivermectin and albendazole can be administered together safely, and there is evidence that they can be combined safely with other medications including azithromycin, praziquantel, and diethylcarbamazine (DEC) [49,50,51].

## 7. Morbidity Management

All of the skin NTDs have the potential to cause chronic ill-health and long-term disability, even after successful treatment of the underlying cause. The patient with treated lepromatous leprosy may have disabling limb deformity and experience recurrent and prolonged episodes of immune-mediated inflammation (erythema nodosum leprosum or type 2 leprosy reaction) for many years after treatment. The patient with treated lymphatic filariasis may suffer with debilitating lymphoedema and repeated episodes of ill-health due to acute dermatolymphangioadenitis (ADLA). The dermatology community can support these patients through interventions that focus on achieving healthy skin and preventing chronic disease complications.

The burden of disease resulting from chronic complications of skin NTDs is poorly understood, and more data are needed to support political advocacy and fundraising. Efforts are being made to address this, for example, through the development of a toolkit for monitoring morbidity and disability across multiple NTDs [52]. The prototype toolkit was validated in Northeast Brazil in patients with Chagas’ disease (American trypanosomiasis), leishmaniasis, leprosy, and schistosomiasis, where it was well accepted.

Community dermatology is the branch of dermatology that addresses skin health at the population level, and this is perhaps most relevant in the poorest communities where the burden of skin disease is high and skin NTDs are prevalent [53,54]. It focuses on providing low-cost interventions for the most common skin diseases, and includes strategies for managing wounds and lymphoedema that are applicable to many skin NTDs.

Simple hygiene-based interventions, typically involving daily washing of affected limbs with soap and water, are known to be effective for the management of lymphoedema in LF-endemic areas, resulting in a reduced incidence of ADLA [55]. Recent data have shown that a low-cost lymphoedema self-care package is effective in reducing the frequency and duration of ADLA in podoconiosis [56]. In this study, the package of care included instructions for foot hygiene, skin care, bandaging, exercises, and use of socks and shoes, and this was reinforced by trained members of the local community at regular monthly meetings. Patients in the treatment group experienced 4.5 fewer episodes of ADLA per person-year compared with the control group who received no intervention.

Lymphatic filariasis (LF) and podoconiosis benefit from a very similar management, and in countries where there is a high burden of both diseases, it is sensible that morbidity management activities should be integrated in order to reduce programme costs and expand coverage. Deribe and colleagues describe the successful integration of these services in Ethiopia [57], where more than 500 government-employed health workers from 24 districts were trained by lymphoedema experts on integrated morbidity management and disability prevention for LF and podoconiosis. Trained health workers follow a management algorithm and deliver a defined package of care to patients, similar to that described earlier [56], focusing on daily hygiene measures, use of emollients to restore skin function, leg elevation, exercise, use of socks and shoes, and bandaging if required. Water, sanitation, and hygiene (WASH) interventions are important for the control of other major NTDs, including trachoma, soil-transmitted helminths, and schistosomiasis [58,59,60], highlighting further possibilities for integrated activities particularly where diseases are co-endemic.

Self-help group interventions are beneficial in many areas of medicine, and could be a useful approach to improve health behaviours for the management of morbidity associated with skin NTDs, with the potential for empowering ‘expert patients’ to take a lead role. For example, women’s groups practising participatory learning and action (PLA) on preventive and care-seeking behaviours are a cost-effective strategy to improve maternal and neonatal survival in low-resource settings [61]. The potential benefits for the skin NTDs are numerous, and range from improved self-care behaviours in the management of lymphoedema to earlier presentation and treatment-seeking in new cases of leprosy in children. A recent study conducted in Nepal showed that the integration of self-care for filarial lymphoedema into existing community leprosy self-help groups was feasible, and attitudes towards integration were positive [62]. Opportunities were identified for LF-affected participants to increase their knowledge of self-care and access to health services.

Stigma and social isolation are a major problem for many NTDs, not least those that have highly visible skin manifestations. The severe stigmatisation of persons affected by leprosy is well described; however, this disability extends to several other skin NTDs including lymphatic filariasis, podoconiosis, Buruli ulcer, onchocerciasis, and leishmaniasis, with similar health-related and psychosocial consequences [63]. Importantly, the reasons for stigmatisation across the different NTDs are remarkably similar; these include appearance, fear of contagion, being a burden to the family, and the inability to fulfil gender roles. It has been suggested, therefore, that integrated approaches to reduce stigmatisation may be feasible and more efficient than disease-specific interventions.

Integrated morbidity management services will also offer an opportunity to promote skin health more broadly. The GBD 2013 data on skin diseases show that dermatitis accounts for the largest global burden, with high DALY rates in many regions of the world, but particularly in central sub-Saharan Africa. The ability to recognise a failing epidermis and prescribe an emollient would be beneficial for many patients, including those with dermatitis and those affected by many of the skin NTDs.

## 8. Conclusions and Future Directions

The grouping together of NTDs that affect the skin draws attention to the significant burden of skin disease that occurs almost exclusively in impoverished populations in the developing world. The concept of skin NTDs is useful for a number of reasons. It facilitates the integration of control activities, from diagnostic processes to community mass drug administration. It creates a strong argument for funding for research and control activities, considering the collective burden of disease. Finally, it places the dermatology community in a strong position to help in the fight against NTDs. Dermatologists can contribute through developing appropriate clinical guidelines, training health workers, coordinating research, and guiding the design and implementation of control activities. This in turn will foster better research relationships internationally and strengthen the fields of tropical and global health dermatology. There is a growing body of literature describing experiences of integration in a variety of settings, offering explanations behind the successes and failures, and it will be important going forward to learn from these experiences. Integrated fieldwork activities, from disease mapping to mass treatment, must be well planned and supported by relevant political bodies and funding agencies. The integrated control of skin NTDs, led by the dermatology community, has the potential to strengthen health systems in resource-poor settings and improve skin health for millions of the world’s most disadvantaged people.
